# Clinical trial considerations on male contraception and collection of pregnancy information from female partner: update

**DOI:** 10.1186/s40169-016-0103-8

**Published:** 2016-07-25

**Authors:** Maria Longauer Banholzer, Christoph Wandel, Paul Barrow, Marie Mannino, Georg Schmitt, Melanie Guérard, Lutz Müller, Gerard Greig, Kenjie Amemiya, Richard Peck, Thomas Singer, Lucette Doessegger

**Affiliations:** 1 Safety Risk Management, Licensing & Early Development, F. Hoffmann-La Roche AG, Basel, Switzerland; 2 Safety Risk Management, Licensing & Early Development, F. Hoffmann-La Roche Ltd, New York, NY USA; 3 Pharma Research & Early Development, Roche Innovation Center Basel, Pharmaceutical Sciences, F. Hoffmann-La Roche AG, Basel, Switzerland; 4 Pharma Research & Early Development, Roche Innovation Center Basel, Clinical Pharmacology, F. Hoffmann-La Roche AG, Basel, Switzerland; 5 Non-Clinical Safety Department, Genentech Inc, South San Francisco, CA USA; 6 Zürich, Switzerland

**Keywords:** Paternal exposure, Genotoxicity, Teratogenicity, Male contraception, Clinical trials

## Abstract

**Background:**

This is an update to our 2012 publication on clinical trial considerations on male contraception and collection of pregnancy information from female partner, after critical review of recent (draft) guidances released by the International Council for Harmonisation [ICH] the Clinical Trial Facilitation Group [CTFG] and the US Food & Drug Administration [FDA].

**Methods:**

Relevant aspects of the new guidance documents are discussed in the context of male contraception and pregnancy reporting from female partner in clinical trials and the approach is updated accordingly.

**Results:**

*Genotoxicity* The concept of a threshold is introduced using acceptable daily intake/permissible daily exposure to define genotoxicity requirements, hence highly effective contraception in order to avoid conception. The duration for highly effective contraception has been extended from 74 to 90 days from the end of relevant systemic exposure. *Teratogenicity* Pharmacokinetic considerations to estimate safety margins have been contextualized with regard to over- and underestimation of the risk of teratogenicity transmitted by a vaginal dose. The duration of male contraception after the last dose takes into account the end of relevant systemic exposure if measured, or a default period of five half-lives after last dose for small molecules and two half-lives for immunoglobulins (mAbs). Measures to prevent exposure of the conceptus via a vaginal dose apply to reproductively competent or vasectomized men, unless measurements fail to detect the compound in seminal fluid.

**Conclusion:**

Critical review of new guidance documents provides a comparison across approaches and resulted in an update of our previous publication. Separate algorithms for small molecules and monoclonal antibodies are proposed to guide the recommendations for contraception for male trial participants and pregnancy reporting from female partners. No male contraception is required if the dose is below a defined threshold for genotoxic concern applicable to small molecules. For men treated with teratogenic mAbs, condom use to prevent exposure of a potentially pregnant partner is unlikely to be recommended because of the minimal female exposure anticipated following a vaginal dose. The proposed safety margins for teratogenicity may evolve with further knowledge.

## Background

In 2012, we proposed an approach to consistently assess need for contraception for men exposed in clinical trials to genotoxic and/or teratogenic compounds or to compounds of unknown teratogenicity, and to collection of pregnancy data from their female partner [1]. The current update was triggered by three guidance documents which became available since 2014, namely, the International Council for Harmonisation (ICH) guideline M7 on mutagenic impurities in drugs [2], the guidance from the Clinical Trial Facilitation Group (CTFG) on recommendations related to contraception and pregnancy testing in clinical trials [3] and the draft guidance for industry from the US Food and Drug Administration (FDA) regarding assessment of male-mediated developmental risk for pharmaceuticals [4].

## Methods

Relevant aspects of the new guidance documents are summarized and the approach to male contraception in clinical trials has been updated accordingly.

## Results and discussion

### Review of relevant aspects from guidance documents

#### ICH M7: assessment and control of DNA reactive (mutagenic) impurities in pharmaceuticals to limit potential carcinogenic risk [2]

Mutations in germ cells can lead to spontaneous abortions, infertility or heritable damage to the offspring and possibly to the subsequent generations [5]. As stated by ICH S2 (R1) [6], “most germ cell mutagens are likely to be detected as genotoxic in somatic cell tests so that negative results of in vivo somatic cell genotoxicity tests generally indicate the absence of germ cell effects”. Thus, standard genotoxicity assays performed in somatic cells in vitro and in vivo are appropriate to assess the risk of potential genetic damage of germ cells.

For genotoxicity, the term threshold is used in two different types of data contexts: (a) to describe non-linear thresholded dose–effect relationships for genotoxic compounds, which mostly interact indirectly with the genetic material; (b) to describe a Threshold of Toxicological Concern (TTC) below which even for a linear dose–effect relationship, the predicted risk is so low that further risk minimization efforts are not warranted.

Non-linear dose–responses suggest that a threshold dose is required in order to induce an adverse effect. Non-linear dose–responses have been reported for low doses of genotoxic compounds with diverse indirect (e.g. aneugens, topoisomerase inhibitors etc.) and direct deoxyribonucleic acid (DNA) reactive mechanisms (e.g. alkylating agents) and the shape of the respective dose–response appears to be dependent on related cytoprotective cellular processes. It is however unlikely that small genotoxic effects, i.e. within the normal background range, can be excluded experimentally. Thus, published data mostly report practical thresholds where non-linear dose–responses are described through statistical approaches to define a point of departure. The least complex are the single effect levels such as the no observed genotoxic effect level (NOGEL) and the lowest observed genotoxic effect level (LOGEL). The regulatory approach to such compounds can be based on the identification of a NOGEL and use of uncertainty factors to calculate a permissible daily exposure when data are available.

For the majority of direct DNA genotoxins there is no experimental evidence of a threshold relative to a genotoxic effect–dose (concentration) curve. In these instances, the dose (concentration)- response curve is linear (i.e. non-thresholded) and the concept of TTC may be applied for risk assessment.

The TTC has originally been allocated for mutagenic impurities in drug formulations at a calculated risk level of one additional cancer case in 100,000 exposed patients for a lifetime intake. The TTC concept was developed to define an acceptable daily intake for any unstudied chemical that poses a negligible risk of carcinogenicity or other toxic effects. The TTC-based acceptable intake of 1.5 μg/day is considered to be protective for a lifetime of daily exposure. To address less-than-lifetime exposure to mutagenic impurities in pharmaceuticals, an approach is applied in which the acceptable cumulative lifetime dose (1.5 μg/day × 25,550 days = 38.3 mg) is uniformly distributed over the total number of exposure days during less-than-lifetime exposure. This would allow a higher daily intake than would be the case for lifetime exposure and still maintain comparable risk levels for daily and non-daily treatment regimens. However, a uniform distribution ignores experimental data, which have demonstrated a higher risk for cancer mediated by mutations if the same cumulative dose is taken over a shorter than a longer duration. Hence, less-than-lifetime exposure values for shorter than lifetime exposure situations have incorporated additional safety margins to account for this experimental observation (Table 1).

**Table 1:** Acceptable intake in relation to less-than-lifetime levels of exposure

	Duration of treatment			
	<1 month	>1–12 months	>1–10 years	>10 years to lifetime
Acceptable daily intake (μg/day)	120	20	10	1.5

#### Clinical trial facilitation group (CTFG): recommendations related to contraception and pregnancy testing in clinical trials [3]

The CTFG supports the Heads of Medicines Agencies, a network of the heads of the National Competent Authorities whose organizations are responsible for the regulation of medicinal products for human and veterinary use in the European Economic Area.

The CTFG guidance document includes the following definitions:

- Highly effective and acceptable contraceptive measures and categorization of contraceptives
- End of relevant systemic exposure: Time point where the Investigational Medicinal Product including any active or major metabolites, has decreased to a concentration that is no longer considered relevant for human teratogenicity/fetotoxicity
- Duration of one sperm cycle: The duration of a sperm cycle is defined as 90 days
- Menopause: A postmenopausal state is defined as no menses for 12 months without an alternative medical cause. A high follicle stimulating hormone (FSH) level in the postmenopausal range may be used to confirm a postmenopausal state in women not using hormonal contraception or hormonal replacement therapy. However in the absence of 12 months of amenorrhea, a single FSH measurement is insufficient.

The key messages of the CTFG guidance paper concerning contraception in context of exposed men in clinical trials are listed below.

- In case of a genotoxic Investigational Medicinal Product, the principle of TTC as available for mutagenic impurities should be considered.
- The following three main risk categories for the early stages of pregnancy have been adapted from the risk categories set in the Committee for Medicinal Products for Human Use (CHMP) guideline EMEA/CHMP/203927/2005 [7]:

Demonstrated or suspected human teratogenicity/fetotoxicity in early pregnancy

Possible human teratogenicity/fetotoxicity in early pregnancy

Unlikely human teratogenicity/fetotoxicity in early pregnancy

- Referring to Klemmt and Scialli [8], CTFG assumes the estimated drug exposure level in the female partner of child-bearing potential after a vaginal dose to be three or more orders of magnitude lower than the plasma concentration in the male clinical trial participant [3]. Subsequently, CTFG concludes that the risk of teratogenicity from a vaginal dose only applies to those drugs with demonstrated or suspected human teratogenicity/fetotoxicity at sub-therapeutic systemic exposure levels and not to drugs with (demonstrated or suspected) teratogenicity at therapeutic or supratherapeutic levels. However, sub-therapeutic systemic exposure level is not further described and quantified.
- In cases where reproductive toxicity studies are available, the anticipated systemic exposure in trial participants should have a sufficient exposure margin below the no adverse effect level (NOAEL) in the non-clinical embryo-fetal development (EFD) studies; otherwise, condom use is required. However, a margin is not quantified.
- In case of insufficient or unavailable non-clinical data, the impact on the risk categorization should be evaluated. “Unavailable or insufficient non-clinical data”, should be considered as “effects detected”, and the highest possible risk category assumed.
- In the absence of EFD studies, end of relevant systemic exposure may be based on the principles of a Minimum Anticipated Biological Effect Level (MABEL) or other accepted principles.

#### FDA draft guidance for industry: assessment of male-mediated developmental risk for pharmaceuticals [4]

The FDA draft guidance for industry was distributed for comments in June 2015. It provides recommendations to sponsors for assessing risks to embryo-fetal development resulting from administration of an active pharmaceutical ingredient to males, either through an effect on the male germ cell or from seminal transfer of an active pharmaceutical ingredient that has been shown to be genotoxic or a potent developmental toxicant. A potent developmental toxicant is defined as one associated with adverse fetal outcomes at or near clinical exposures or for which a NOAEL has not been defined.

The guidance does not consider the situation where no EFD studies were performed and does not specify safety margins. Examples are provided with estimated exposure multiples of 50- fold and 20-fold between C_max_ in female partners and C_max_ associated with NOAEL in EFD studies with the conclusion that no further assessment of the drug in seminal fluid would be needed. Examples for no NOAEL from EFD studies are not provided.

### Risk assessment considerations

#### Risk assessment for genotoxicity

The impact of positive genotoxicity findings on the development of a small molecule depends on the indication, the patient population and the duration of treatment. Genotoxic compounds are generally not tested in healthy volunteers and if so, require an extensive risk assessment. According to literature, in general, single-dose clinical studies are permitted by the FDA regardless of the genotoxicity test results [9]. However, some other Health Authorities may not agree to test genotoxic compounds with single doses in healthy volunteers or may request a rationale to do so and safety margins have been accepted on a case-by-case basis.

The existence of mechanisms leading to a dose response that is non-linear and has a practical threshold is increasingly recognized, not only for compounds that interact with non-DNA targets but also for DNA-reactive compounds [10]. Those effects may be modulated, for example, by rapid detoxification before coming into contact with DNA, or by effective repair of induced DNA lesions. Direct DNA interactions may result in the formation of small or bulky adducts, DNA strand cross-links, DNA–protein cross-links and DNA strand breaks. Indirect mechanisms can be related to the inhibition of DNA repair, impairment of chromosome segregation, disruption of mitotic checkpoints machinery, inhibition of apoptosis, perturbation of cytokinesis, inhibition of enzymes involved in the maintenance of DNA methylation, and induction of inflammation and/or mitochondrial dysfunction leading to increased oxidative stress [11].

For drugs with an indirect (non-DNA) genotoxic mechanism, a non-linear genotoxic effect to dose (concentration) curve is typical, allowing the identification of a NOGEL. Based on the integrated data, the genotoxic risk and the safety margin need to be assessed, including the cut-off concentration or permissible daily exposure in the female partner below which the predicted genotoxic risk is low, hence, for which highly effective contraception including condom use is not needed.

Also for some molecules with a direct genotoxic mechanism, a non-linear dose genotoxic response with a range of non-mutagenic low concentrations and a NOGEL has been found in vitro, as it is typically seen for molecules with an indirect mode of genotoxicity [12, 13]. This is likely based on specific DNA lesions and their removal by DNA repair enzymes in an error-free fashion. This process cannot be generalized and for the majority of direct DNA genotoxins there is no experimental evidence of a threshold relative to a genotoxic effect–dose (concentration) curve. In these instances, the dose (concentration) response curve is linear (i.e. non-thresholded) and the concept of TTC as defined for impurities in ICH M7 [2] may be applied for risk assessment.

#### Implications of guidance documents on the 2012 genotoxicity approach

In comparison to the 2012 publication [1], the concept of a threshold in line with ICH M7 is introduced using acceptable daily intake or permissible daily exposure to define genotoxicity requirements. However, application of the TTC concept with acceptable daily intake, adopted from CTFG, very likely results in recommending highly effective contraception based on the very low acceptable daily intake. For methods of highly effective contraception, reference is made to the CTFG guidance document as recommended by MHRA. The MHRA Medical Guidance on “Clarification of contraceptive wording in clinical trials”, 2009 is superseded. Also, the duration of highly effective contraception has been extended from 74 to 90 days from the end of relevant systemic exposure as suggested by the CTFG guidance document. Finally, a reference has been added supporting testing of single doses in clinical trials in the US, regardless of genotoxicity test results.

#### Risk assessment for teratogenicity

*Pharmacokinetics (PK) considerations*

**Scenario 1: A NOAEL from EFD studies in animals is available**

*Preclinical reference parameters* In this scenario the preclinical pharmacokinetic reference parameter taken for safety margin calculations is the Area Under the Curve (AUC) associated with the NOAEL from the more sensitive animal species.

*Clinical reference parameters/estimating the exposure in the female partner after a vaginal dose* The AUC estimated for the female parameter following a vaginal dose is used as reference for comparison against the AUC associated with the NOAEL from EFD animal studies.

Indeed, drug induced teratogenicity in animals has been found to be associated with maximum concentration (C_max_) or AUC [14]. Because the ratio of C_max_/AUC in small animals as used in EFD studies is typically higher than in man, using AUC instead of using C_max_ as the relevant PK parameter provides a smaller margin between exposure in animals and the estimated exposure in female partner. In consequence, calculating safety margins based on AUC represents the more conservative approach compared to safety margins calculated with C_max_.

Small molecules: The dose-exposure relationship for plasma concentrations is assumed to be similar for the female partner and the exposed male. Exposure parameters, here AUC, in the female partner are extrapolated for the vaginal dose from available clinical PK data or PK modeling assuming linear PK.

The vaginal dose is derived from multiplying the seminal concentration with the volume of seminal fluid (6 mL corresponds to the 90 percentile for seminal volume [15]). The seminal concentration is assumed to equal the plasma concentration in the exposed male. The extent of vaginal absorption and the placental transfer relative to female exposure are assumed to be 100 % each.

Monoclonal antibodies (mAbs): For mAbs, the calculation of the vaginal dose assumes a seminal concentration of 1 % of the plasma concentration because this is the ratio commonly reported for IgGs [16–18]. In a recent publication on an IgG2, a 2 % ratio for seminal fluid/plasma concentration was measured [19]. The extent of vaginal absorption is assumed as 10 % given the limited information available [20, 21]. However, a recent publication in Cynomolgus monkeys shows an extent of IgG vaginal absorption well below 1 % [22, 23].

A dilution factor of 500 is used on the basis that the seminal fluid volume is 6 mL and the plasma volume is about 3000 mL which typically represents the volume of distribution of the mAb. Overall, this estimate results in the assumption that the concentrations of mAbs in the plasma of the female partner is ~500,000-times lower than in the exposed male trial participant:  ×100 (1 % seminal concentration relative to plasma concentration in exposed male) ×10 (10 % due to vaginal absorption) ×500 (dilution of 6 mL seminal fluid in plasma volume of 3000 mL).

**Scenario 2: No NOAEL from EFD studies in animals has been established or no EFD studies are available**

*Preclinical reference parameters* In this scenario, the MABEL derived from an AUC in animal studies or representing a concentration resulting from a cellular experiment may be considered.

*Clinical reference parameters* In case the MABEL represents an in vitro concentration, the estimated C_max_ for the female partner after a vaginal dose should be used. In case the MABEL relates to an animal experiment, we propose to compare the associated AUC with the estimated AUC in the female partner. Please note, the use of AUC was not specified in the 2012 publication. The approach for estimating the exposure in female partner is outlined for scenario 1.

The MABEL approach should consider additional points:

- No off-target effect including (major) metabolites.
- A MABEL assessment in an in vitro setting may refer to a target outside the plasma compartment, e.g. the brain. PK considerations should then translate the MABEL concentration to the associated predicted concentrations in the plasma compartment. Margin calculations use the estimated female exposure, here C_max_, and the relevant plasma concentration corresponding to the MABEL.
- No toxicities linked to pharmaceutical properties (e.g. drug deposition in bradytrophic tissues such as bone, cartilage, lens).
- The MABEL derived exposures are lower than the lowest exposure tested in EFD studies.

**First pass effects and underestimation of exposure to female partner and embryo/fetus**

The mode of estimating the exposure in the female partner after a vaginal dose may result in underestimation of the exposure in the female partner and in the embryo/fetus. For small molecules administered vaginally this may include the absence of intestinal-hepatic first pass effect and the presence of uterine first pass effect. The latter may also be applicable for mAbs. In case an estimate for a first pass effect is available, the safety margin may be individualized.

*Intestinal*-*hepatic first pass effect* The extrapolation from vaginal dose to exposure in female partner refers to available PK information. If this PK information is derived from PK after oral administration and if the molecule undergoes relevant first pass effect by intestine and liver, the estimated female exposure after vaginal dose will underestimate the true exposure because of the absence of first pass intestine and hepatic effects after the vaginal route of administration. The absence of the intestinal-hepatic first pass effect after a vaginal dose may be corrected by multiplying the derived estimated AUC/C_max_ values from oral administration with [1 ÷ (1 − first pass effect)].

*Uterine first pass effect* The uterine first pass effect describes the extraction of drug by the uterus following the countercurrent transfer of a molecule after vaginal absorption via vaginal vein drainage to uterine arteries. When measured in non-pregnant subjects or in perfused hysterectomy, the ratio of drug concentration in endometrium or uterine artery versus systemic vein concentration is about two to sevenfold higher after vaginal than after systemic drug administration based on reports for progesterone [24], danazol [25], thalidomide [26].

*Safety margin considerations*

Regulatory guidelines lack recommendations regarding safety margins for teratogenicity between NOAEL in EFD studies a and maximum doses tested in clinical trials. For first-in-human studies applying a range of escalating doses, the FDA suggests a default safety factor of ten between the exposure for the starting dose and that of the Human Equivalent Dose, which should be individualized [27]. In our experience, for non-monitorable and potentially irreversible and severe toxicities, a safety factor of ten is usually applied between exposure associated with the maximum dose in a clinical trial and the NOAEL in EFD studies. Given the uncertainties around predicting exposure in the female partner/fetus and the potential serious outcome in the development of the embryo-fetus, a safety margin higher than tenfold is proposed between NOAEL in EFD studies and exposures associated with the maximum dose in the clinical trial. Of note, the FDA draft guidance states that a margin of at least tenfold between NOAEL in EFD studies and the estimated exposure to embryo-fetus due to vaginal dose would not mandate evaluation of drug in seminal fluid.

**Scenario 1: safety margins proposed when a NOAEL from EFD studies in animals is available**

When a NOAEL and its associated AUC from non-clinical EFD studies is available, a margin of at least 300 is requested relative to the expected exposure (AUC) in the female partner for small molecules and of at least 100 for mAbs. The smaller margin for mAbs appears justified because the exposure in the female partner is more predictable than for small molecules, given the more generalizable PK of mAbs. If the seminal concentration for a small molecule is not estimated, but has been measured, we propose to reduce the required safety margin from 300 to 100.

**Scenario 2: safety margins proposed when no NOAEL Has been established in EFD studies or no EFD studies are available**

When a NOAEL has not been established or no EFD studies have been conducted, male contraception is proposed unless there is a sufficient margin between the estimated exposure of female partner and the MABEL of the molecule.

Margin calculations using MABEL refer to the distinction between teratogenicity at therapeutic and sub-therapeutic levels as made in the CTFG paper. For a Hill coefficient e.g. of 1, a 81-fold increase in concentration is needed to increase a response from 10 (assumed level for MABEL) to 90 % (assumed therapeutic level) [28]. In the safety margin considerations below, a factor of 100 instead of 81 is considered between MABEL, taken as sub-therapeutic level, and therapeutic level.

For small molecules, the safety margins as defined for EFD studies with NOAEL apply, i.e. 300 when the seminal concentration is estimated and 100 if the seminal concentration is measured.

For mAbs, the safety margin from EFD studies with NOAEL is lowered from 100 to 10 with the MABEL approach to account for the limited placental transfer of about 10 % during the most vulnerable organogenesis phase, i.e. the first trimester.

**Fig. 1:**
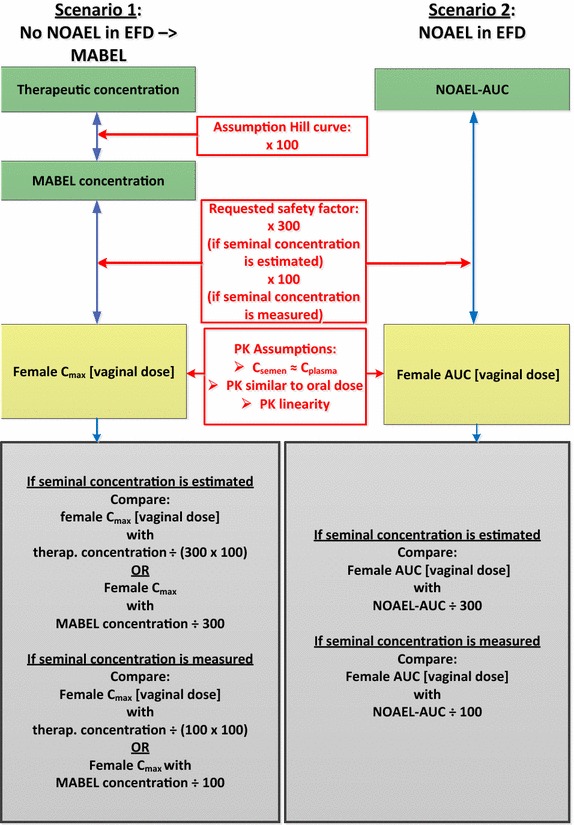
Safety margin estimations: small molecules. *AUC* area under the curve, *C*_*max*_ maximum concentration, *C*_*plasma*_ plasma concentration, *C*_*semen*_ seminal concentration, *EFD* embryo-fetal development, *MABEL* minimum anticipated biological effect level, *NOAEL* no adverse effect level

**Fig. 2:**
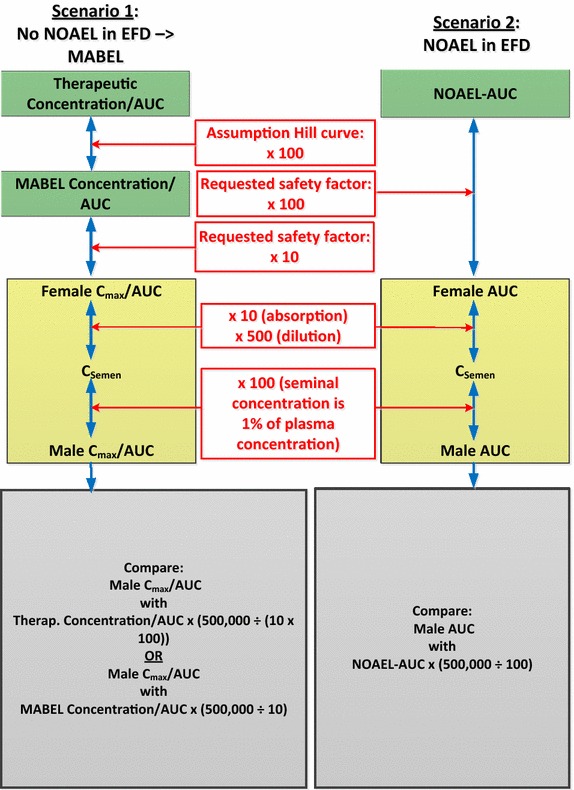
Safety margin estimations: monoclonal antibodies. *AUC* area under the curve, *C*_*max*_ maximum concentration, *C*_*semen*_ seminal concentration, *EFD* embryo-fetal development, *mAb* monoclonal antibody, *MABEL* minimum anticipated biological effect level, *NOAEL* no adverse effect level

The safety margin estimations supporting contraceptive recommendations are summarized in Fig. 1 for small molecules and in Fig. 2 for mAbs.

#### Differences in PK and safety margin considerations between our approach and the FDA draft guidance

Comparison is done to the draft FDA guidance since the CTFG guidance does not include details about PK.

Factors used to estimate the exposure in the female partner after a vaginal dose for small molecules and mAbs are displayed in Table 2 which includes a comparison with the FDA draft guidance.

**Table 2:** Factors to estimate the exposure in the female partner after a vaginal dose: comparison with the approach from the fda draft guidance

	Updated proposal		FDA draft guidance	
	Small molecules	mAbs	Small molecules	mAbs
Pre-clinical reference model and PK parameter for safety margin calculations				
Pre-clinical reference model	NOAEL from EFD studies or MABEL if no NOAEL from EFD studies is available		NOAEL from EFD studies, no reference to MABEL	
PK parameter	AUC if NOAEL from EFD studies is available or if MABEL refers to AUC; C_max_ if MABEL refers to a concentration		C_max_	
Estimating exposure in female partner				
Ejaculation volume	6 mL		5 mL	
Seminal concentration	Total C_max_ in plasma	1 % of total C_max_ in plasma	Total C_max_ in plasma	1 % of total C_max_ in plasma
Vaginal absorption	100 %	10 %	100 %	10 %
Estimating exposure in female partner relative to vaginal dose absorbed	Extrapolates AUC and C_max_ in female partner relative to the vaginal dose absorbed based on available dose-exposure	AUC of exposed male divided by 100 (ratio seminal fluid concentration: plasma concentration), by 10 (vaginal uptake), by 500 (ratio plasma volume: seminal fluid volume)	Estimates C_max_ by dividing vaginal dose absorbed by blood volume (5000 mL) *Note: For mAbs the estimated C*_*max*_ *in the female partner may be underestimated as the volume of distribution typically is the plasma* *For small molecules, the estimated female C*_*max*_ *is likely to result in an overestimation as vaginal dose divided by blood volume assumes an absorption rate similar to rapid iv injection. Indeed, pharmacokinetic data for drugs administered vaginally show that T*_*max*_ *is not instant as per IV administration but takes several hours*	
Vaginal administration: no intestinal-hepatic first pass effect	An intestinal-hepatic first pass effect would be assumed if vaginal administration is extrapolated from oral PK data, resulting in a possible underestimation of exposure to embryo/fetus.		No intestinal-hepatic first pass effect is taken into account. Thus, there is no potential for underestimation of exposure to embryo/fetus	
Placental transfer	100 %	10 % in 1st trimester; 100 % in 3rd trimester	100 %	10 % in 1st trimester; 100 % in 3rd trimester
Uterine first pass effect	Not taken into consideration, potential underestimation of exposure to embryo/fetus			
Safety margin	300, when seminal concentration is estimated; 100, when seminal fluid concentration of molecule is known	100 10, when referring to MABEL	Not specified	

*AUC* area under the curve, *C*_*max*_ maximum concentration, *EFD* embryo-fetal development, *IV* intravenous, *mAb* monoclonal antibody, *MABEL* minimum anticipated biological effect level, *mL* milliliter, *NOAEL* no adverse effect level, *PK* pharmacokinetics

To calculate the vaginal dose, the FDA draft guidance uses a seminal fluid volume of 5 mL instead of 6 mL (6 mL corresponds to the 90 percentile for seminal volume [15]).

For mAbs, the FDA approach estimates C_max_ in female partner as vaginal dose absorbed divided by blood volume instead of plasma volume. Consequently, the dilution factor to estimate female exposure from vaginal dose is half in our approach compared with the one suggested in the FDA draft guidance. Because the PK derived from animals typically refers to measurements of mAbs in plasma, it is therefore important to ensure that exposure comparisons between species apply the same matrix and compartment.

For small molecules, the FDA approach estimates the female C_max_ as vaginal dose divided by blood volume which corresponds to C_max_ after rapid iv injection. Given the delayed time to maximum plasma concentration after vaginal administration this overestimates C_max_. Indeed, PK data for drugs administered vaginally show that the time to maximum plasma concentration is not instant as per IV administration but takes several hours, e.g. for metronidazole [29], clindamycin [30] and progesterone [24]. It is acknowledged that the vaginal absorption rate is dependent on the physico-chemical properties of the molecule, the formulation used, and the vaginal epithelium status. The C_max_ in female partners is influenced by the volume of distribution, which varies across small molecules. Consequently, C_max_ is likely lower compared to the estimate as suggested in the FDA draft guidance.

In our approach, the presence of an intestinal-hepatic first pass effect, when using oral PK data to extrapolate from vaginal dose to female systemic exposure, results in an underestimation of exposure to female partner and embryo/fetus. As stated previously, in case an estimate for a first pass effect is available, the safety margin may be individualized. The FDA approach is not associated with such an underestimation because this approach does not refer to available PK data after oral administration when estimating female exposure after vaginal route of administration.

On the other hand and as outlined earlier, using AUC rather than C_max_ to compare NOAEL in animals with estimated female exposure represents the more conservative selection of PK parameter.

#### Implications of guidance documents on the 2012 teratogenicity approach

The CTFG makes reference to sub-therapeutic and therapeutic levels for assessing the need for contraception. Furthermore, the guidance suggests the MABEL concept as a preclinical reference parameter in situations where no EFD studies are available to define the end of relevant systemic exposure. In our 2012 publication, the use of MABEL has been suggested in the case no EFD studies are available or no NOAEL could be established in EFD studies in order to estimate safety margins whereby MABEL is considered to be a sub-therapeutic level. The examples provided in the FDA draft guidance do not include MABEL or a scenario with no NOAEL in EFD studies. The MABEL recommendation as specified in our 2012 publication remains unchanged.

The FDA draft guidance as well as our approach suggest that condom use is recommended in case a teratogenic risk remains unknown/uncertain or in case a risk of male-mediated developmental toxicity has been determined to exist. In the CTFG guidance document, the contraception recommendations also apply for risk unknown. However, condom use for embryo-fetal risk from treatment of male subjects with investigational medicinal products only applies to investigational medicinal products with demonstrated or suspected human teratogenicity/fetotoxicity in early pregnancy at sub-therapeutic systemic exposure levels (not further specified). The recommendation for condom use in case of teratogenicity remains unchanged.

The FDA draft guidance emphasizes the need for condom use also for vasectomized men unless measurements using an adequate assay fail to detect the molecule in seminal fluid. This consideration has been added to our approach.

### Contraceptive needs

#### Contraceptive needs relative to genotoxicity testing outcome

With genotoxic small molecules, the aim is to avoid conception until the end of relevant systemic exposure plus 90 days. Men with a female partner of childbearing potential should use condoms plus an additional contraceptive method that together result in a failure rate of <1 % per year if the criteria below are met:

- The small molecule has been found to be genotoxic in non-clinical studies or genotoxicity testing has not been completed
- The clinical dose is >permissible daily exposure/acceptable daily intake based on NOGEL for compounds with a thresholded non-linear dose–response relationship or based on TTC for compounds with a linear dose–response relationship, respectively.

*Duration of contraceptive measures*

The contraceptive measures should be maintained during treatment and until the end of relevant systemic exposure plus 90 days (i.e. 60–75 days for sperm production plus 10–14 days for the transport to the epididymis). In general, the end of relevant systemic exposure for small molecules may be defined, by default, as five half-lives after the last dose.

#### Contraceptive needs relative to teratogenicity testing outcome

With teratogenic compounds, the aim is to avoid exposure of the conceptus via a vaginal dose until the end of relevant systemic exposure.

*Small molecules*

Condom use in men with a pregnant partner or a partner of child-bearing potential (i.e. unknown early pregnancy status) is required under the following circumstances:

Scenario 1: NOAEL from EFD studies is available

- Female AUC extrapolated from the vaginal dose (male C_max_ × 6 mL of semen) > (NOAEL AUC) ÷ 300 (if seminal concentration is estimated) or
- Female AUC extrapolated from the vaginal dose (male C_max_ × 6 mL of semen) > (NOAEL AUC) ÷ 100 (if seminal concentration has been measured)

Scenario 2: EFD or NOAEL from EFD studies is not available

- Female C_max_ or AUC extrapolated from vaginal dose (male C_max_ × 6 mL of semen) > (MABEL concentration or AUC) ÷ 300 (if seminal concentration is estimated) or
- Female C_max_ or AUC extrapolated from vaginal dose (male C_max_ × 6 mL of semen) > (MABEL concentration or AUC) ÷ 100 (if seminal concentration has been measured) or
- Female C_max_ or AUC extrapolated from vaginal dose (male C_max_ × 6 mL of semen) > (therapeutic concentration or AUC; in case Hill coefficient is 1) ÷ 30,000 (if seminal concentration is estimated) or
- Female C_max_ or AUC extrapolated from vaginal dose (male C_max_ × 6 mL of semen) > (therapeutic concentration or AUC; in case Hill coefficient is 1) ÷ 10,000 (if seminal concentration has been measured)

*Monoclonal antibodies*

Condom use in men with a pregnant partner or a partner of child-bearing potential (i.e. unknown early pregnancy status), is required under the following circumstances:

Scenario 1: A NOAEL from EFD studies is available

- Male AUC > (NOAEL AUC) × 5000

Scenario 2: NOAEL from EFD studies is not available

- Male C_max_ or AUC > (MABEL concentration or AUC) × 50,000
- Male C_max_ or AUC > (therapeutic concentration or AUC) × 500

*Duration of condom use*

Condom use is required during treatment and until the end of relevant systemic exposure, which may be defined by default, as five half-lives after the last dose for small molecules and two half-lives after the last dose for mAbs.

#### Combined algorithms for genotoxicity and teratogenicity

**Fig. 3:**
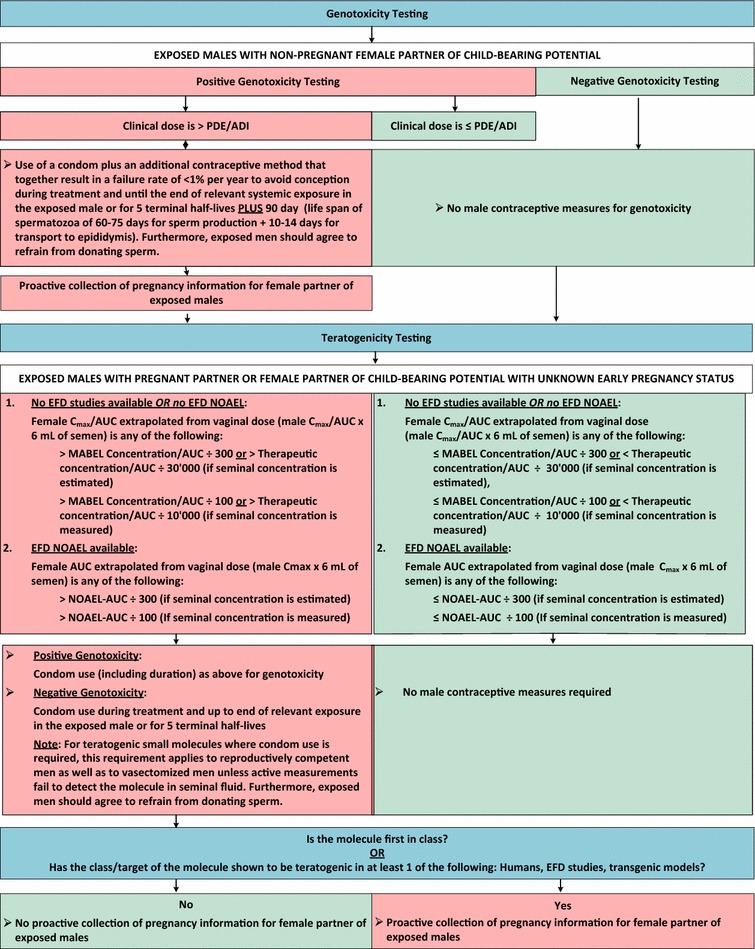
Algorithm for small molecules. *ADI* acceptable daily intake, *AUC* area under the curve, *C*_*max*_ maximum concentration, *EFD* embryo-fetal development, *MABEL* minimum anticipated biological effect level, *NOAEL* no adverse effect level, *PDE* permissible daily exposure

**Fig. 4:**
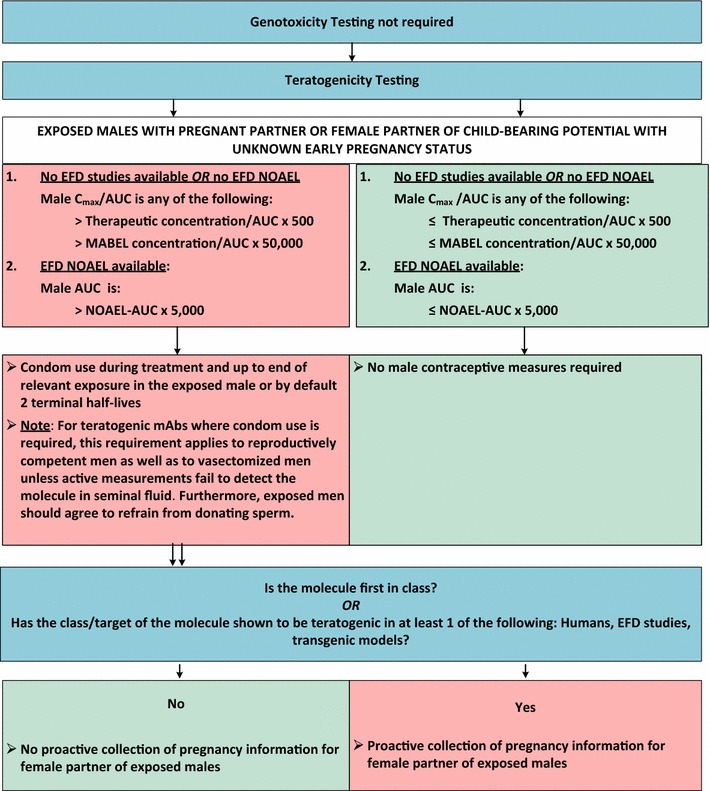
Algorithm for monoclonal antibodies. *AUC* area under the curve, *C*_*max*_ maximum concentration, *EFD* embryo-fetal development, *mAb* monoclonal antibody, *MABEL* minimum anticipated biological effect level, *NOAEL* no adverse effect level

Figures 3 and 4 show algorithms combining genotoxicity and teratogenicity for small molecules and for mAbs, respectively.

## Conclusions

Relevant aspects of new guidance documents were taken into account in updating our approach to male contraception requirements in clinical trials.

For genotoxicity, a threshold concept based on ICH M7 and in line with CTFG, was introduced using permissible daily exposure/acceptable daily intake to define the requirement for highly effective contraception related to small molecules. The use of acceptable daily intake based on a TTC is unlikely to result in a waiver for highly effective contraception because the clinical daily dose of a molecule to be tested must be very low (e.g. up to 120 µg per day for a treatment period up to 1 month). Hence, the impact of using a TTC on the 2012 contraception recommendations is considered low.

For men treated with teratogenic mAbs, condom use to prevent exposure of a potentially pregnant partner is unlikely to be recommended because of the minimal female exposure anticipated following a vaginal dose.

Pharmacokinetic considerations for estimating exposure in female partners after vaginal dose are compared across the CTFG guidance document, the FDA draft guidance, and our approach. In particular with regard to the potential for over- or underestimation of the exposure in female partners and embryo-fetus. Outline of these aspects contributes to the proposal for safety margins and potential individualization relative to PK characteristics. The proposed safety margins for teratogenicity may evolve with further knowledge and interactions with Health Authorities. Finally, new methods of contraception may become available over time.

Since female partners do not participate in the clinical trial and have not given their consent, it is the responsibility of the man exposed to a study drug to inform his female partner of the need to comply with the risk minimization measures. Pregnancy data should be proactively collected for females becoming pregnant as partner of men given genotoxic small molecules. Similarly, data should be collected if the female partner becomes pregnant during the period condom use is required for men exposed to molecules which are first-in-class or with a target/class shown to be teratogenic, embryotoxic or fetotoxic in human or in preclinical experiments. Recommendations for collection of pregnancy information from female partners of exposed men are unchanged compared to our 2012 publication.

This work represents the view of the authors based on current knowledge after consideration of new aspects relevant to assess the potential genotoxic and/or teratogenic risk via a seminal dose.

## Abbreviations

AUC: area under the curve
C_max_: maximum concentration
CTFG: Clinical Trial Facilitation Group
DNA: deoxyribonucleic acid
EFD: embryo-fetal development
FDA: Food & Drug administration
ICH: International Council for Harmonization
mAb: monoclonal antibody
MABEL: minimum anticipated biological effect level
NOAEL: no adverse effect level
NOGEL: no genotoxic effect level
PK: pharmacokinetics
TTC: threshold of toxicological concern
