# Erratum to: Clinical trial considerations on male contraception and collection of pregnancy information from female partner: update

**DOI:** 10.1186/s40169-016-0115-4

**Published:** 2016-08-12

**Authors:** Maria Longauer Banholzer, Christoph Wandel, Paul Barrow, Marie Mannino, Georg Schmitt, Melanie Guérard, Lutz Müller, Gerard Greig, Kenjie Amemiya, Richard Peck, Thomas Singer, Lucette Doessegger

**Affiliations:** 1 Safety Risk Management, Licensing & Early Development, F. Hoffmann-La Roche AG, Basel, Switzerland; 2 Safety Risk Management, Licensing & Early Development, F. Hoffmann-La Roche Ltd, New York, NY USA; 3 Pharma Research & Early Development, Roche Innovation Center Basel, Pharmaceutical Sciences, F. Hoffmann-La Roche AG, Basel, Switzerland; 4 Pharma Research & Early Development, Roche Innovation Center Basel, Clinical Pharmacology, F. Hoffmann-La Roche AG, Basel, Switzerland; 5 Non-Clinical Safety Department, Genentech Inc, South San Francisco, CA USA; 6 Zurich, Switzerland

## Erratum to: Clin Trans Med (2016) 5:23 DOI 10.1186/s40169-016-0103-8

Following publication of the original article in* Clinical and Translational Medicine* [1], it was brought to our attention that there is an error in the section “Safety margin considerations”.

The second sentence of the first paragraph of the section “Safety margin considerations” currently reads: “For first-in-human studies applying a range of escalating doses, the FDA suggests a default safety factor of ten between the exposure for the starting dose and that of the Human Equivalent Dose, which should be individualized [27]”.

This text should read: For first-in-human studies applying a range of escalating doses, the FDA suggests a default safety factor of ten between the NOAEL in GLP toxicology studies in animals and the starting dose expressed as Human Equivalent Dose of the first-in-human study. The safety margin should be individualized by compound [27].
